# Deciphering patient selection of physicians in online health communities: insights from a dual-path perspective

**DOI:** 10.1186/s40359-025-03796-5

**Published:** 2025-12-26

**Authors:** Chengyi Le, Jinping Zhang, Zixin Wang, Feiyan Qiu

**Affiliations:** 1 School of Bussiness, Ningbo University, Ningbo, 315211 China; 2 Merchants’ Guild Economics and Cultural Intelligent Computing Laboratory, Ningbo University, Ningbo, 315211 China; 3 School of Economics and Management, East China Jiaotong University, Nanchang, 330013 China

**Keywords:** Online health community, Online medical choice behaviour, Dual-route model, User involvement, Online trust

## Abstract

**Objectives:**

Online medical choice behaviour in online health communities represents a key step in converting online consultation services. Understanding its influencing mechanisms offers practical value for improving user experience and platform operations. Prior studies mainly examined single information elements and lacked a systematic analysis of multiple information-processing routes and their mechanisms.

**Methods:**

We used data from 313 physicians in the psychiatry and psychology department of an online health community (Haodf.com) in China. Guided by the Elaboration Likelihood Model and trust theory, we built regression models incorporating the central route (content quality and interaction quality) and the peripheral route (reputation credibility, professional competence credibility, and service capability credibility). We tested the effects of these information factors on patients’ online trust and online medical choice behaviour, and further examined the moderating roles of user involvement and consultation price.

**Results:**

Content quality, interaction quality in the central route, and reputation and service capability credibility in the peripheral route significantly enhanced patients’ online trust. Online trust, in turn, significantly positively affected online medical choice behaviour. Moreover, higher user involvement strengthened the impact of central route factors on online trust, while higher consultation price weakened the positive effect of online trust on online medical choice behaviour.

**Conclusions:**

This study demonstrates the differentiated roles of multiple information-processing routes in shaping trust and behavioural transformation in online health communities. The findings provide empirical evidence to support optimised interface design and differentiated service strategies for online consultation platforms.

## Lay summaries

Understanding what drives patients to choose physicians in online health communities is critical for improving consultation conversion rates. Our study shows that the quality and interactivity of physicians’ content (central route) and the credibility of their reputation and service capability (peripheral route) play essential roles in shaping online trust. In turn, online trust strongly promotes patients’ medical choice behaviour. We also found that user involvement amplifies the influence of central route factors on online trust. At the same time, a higher consultation price reduces the positive impact of online trust on medical choice. These findings highlight how online platforms can optimise interface design and service strategies to better support patient decision-making.

## Introduction

Online health communities represent an essential form of digitalizing medical resources and are increasingly becoming a primary channel for patients to seek medical advice. Unlike traditional offline visits, patients in online health communities make decisions through online medical choice, a process in which they select physicians for consultation based on multidimensional information provided by the platform. The core indicator of this process is usually the consultation conversion rate [1]. As health demands have grown and internet healthcare infrastructure has improved, online medical choice behaviour has shown a rapid upward trend in recent years. For example, data from the China Internet Network Information Centre (CNNIC) indicated that as of June 2023, the number of online medical users in China reached 364 million, accounting for 33.8% of all internet users, with an increase of 1.62 million compared with December 2022 [2]. Another nationwide retrospective study confirmed that the number of online consultations in China has grown over the past decade and has gradually become an important way for patients to access healthcare services [3]. This background highlights the practical significance of exploring the mechanisms of patients’ decision-making in online health communities.

Research in health information behaviour has achieved considerable progress [4], but studies focusing on online medical choice still have notable limitations. On the one hand, most studies emphasize single information elements, such as consultation volume or number of comments, and lack a systematic analysis of how patients process and use multidimensional information [5]. On the other hand, although empirical studies have revealed significant influences of word of mouth, service quality, and physician characteristics on medical choice [6–8], explanations of the underlying psychological mechanisms remain insufficient, especially concerning how information transforms into actual decision-making. In this process, the abundant physician–patient interaction information in online health communities offers patients valuable references [9]. Such information enhances patient participation in the medical service process and facilitates their gradual adaptation to and trust in online consultation models.

To deepen understanding of this process, the Elaboration Likelihood Model(ELM) provides a strong theoretical basis. According to this model, the central and peripheral routes influence attitude formation and change [10].In online health communities, patients may systematically process through the central route, evaluating physicians’ content quality (such as readability of replies and information amount in replies) and interaction quality (such as communication frequency and response timeliness). Alternatively, they may rely on heuristic cues through the peripheral route, such as physicians’ reputation credibility, professional competence credibility, or others’ evaluations, to make quick judgments. The Elaboration Likelihood Model thus offers a solid framework for explaining differences in information processing during medical choice.

However, the Elaboration Likelihood Model alone cannot fully explain how patients identify valuable information in complex environments. Because of the high professionalism of medical services and information asymmetry, patients often find it challenging to assess physicians’ capabilities directly. Patients can regard factors such as physicians’ qualifications, interaction behaviours, and patient feedback on the platform as actively or passively released signals that help them judge physicians’ ability and reliability under uncertainty [11]. Signalling theory complements the Elaboration Likelihood Model by revealing which information serves as credible references in patients’ medical choice processes.

Trust emerges as the key mechanism linking information to behaviour in this context. Whether patients process high-quality information through the central route or capture reputation cues through the peripheral route, such information must translate into online trust before influencing medical choice. In high-risk medical contexts with information asymmetry, online trust reduces patients’ uncertainty and promotes their transition from browsing to actual paid consultations [12, 13].

Based on this understanding, we chose Haodf.com, China’s largest online health community, as the research context. Combining the Elaboration Likelihood Model with signalling theory, we constructed a dual-route framework for patients’ online medical choice to systematically test how central route and peripheral route information influence online trust and online medical choice behaviour. Furthermore, we incorporated two critical moderators, user involvement and consultation price, to explore boundary conditions in the decision-making process. This study aims to address the following questions:

- RQ1: In online health communities, which types of information influence patients’ online trust and online medical choice behaviour? Do different information routes exert differentiated effects?
- RQ2: Do user involvement and consultation price moderate the effects of information on patients’ medical choice decisions?

## Related literature and theory

### Online health communities

Online health communities serve as core platforms in digital healthcare, and current research mainly focuses on three dimensions: platform functions, information content, and user behaviour. Studies on platform functions examine operational models and service mechanisms of online health communities [14, 15]. Research on information content investigates the characteristics of health needs [16, 17], structural attributes of users [18, 19], and classifications of health-information users [20, 21]. Research on user behaviour covers multiple aspects, including search and browsing [22], switching behaviour [23, 24], physician–patient interaction [25, 26], evaluation behaviour [27, 28], adoption behaviour [6, 29], and decision-making behaviour [30]. From the perspective of a full participation path, the use process in online health communities includes five stages: generation of health needs → platform visit → information browsing → physician selection → service feedback. The physician-selection (medical choice) stage connects information acquisition with service consumption and directly influences patients’ care experiences and health outcomes. However, existing studies mainly focus on information search and adoption behaviour, and provide relatively limited discussion of the physician-choice process, especially the systematic analysis of how patients process complex medical information and make decisions.

### Online medical choice behaviour

Research on patients’ medical choice behaviour in online health communities has developed several lines of research. First, physicians’ online word of mouth and service quality are major influencing factors [31, 32]. Liu and Ye [33], drawing on service quality theory and considering both treatment effectiveness and service attitude as well as the moderating role of disease risk, analysed how online word of mouth shapes patients’ medical choice. Xu Xiaoting et al. [7] found that indicators used to measure physicians’ online service quality—including treatment effectiveness, service attitude, number of thank-you letters, number of comments, and the sentiment of comments—significantly and positively influence patients’ choices. Second, the quality of physician–patient interaction matters in medical choice. Liu et al. [34], using data on 4,434 physicians treating chronic diseases, reported that breadth and length of interaction significantly attract patients.

In contrast, greater interaction depth reduces the probability of being chosen, revealing interaction’s complexity and multi-dimensional nature. Third, scholars have widely recognised the pivotal role of trust in physician–patient relationships. Severe information asymmetry between physicians and patients makes trust a key mechanism influencing patients’ decisions to use online medical services [8, 35]. Zeng Yuying et al. [36] and Wu Jiang et al. [37] examined how provider personality, trust-transfer factors, and reputation affect patients’ purchase decisions for online health services. Gong [38] found that physician quality and reputation significantly influence patients’ medical choices and that physician gender can strengthen this influence.

Although prior work has highlighted the importance of word of mouth, service quality, interaction, and trust, two limitations remain. First, many studies treat information effects as a single route and overlook the possibility that patients may adopt dual information processing mechanisms. Second, while acknowledging the role of trust, prior studies seldom explore how different types of information transform into medical choices via the mechanism of trust.

### Elaboration likelihood model

The Elaboration Likelihood Model proposed by Petty and Cacioppo in 1986 [10] explains attitude formation and change through two processes. Individuals may follow a central or peripheral route when processing persuasive information. The central route relies on in-depth reflection and logical evaluation and requires higher cognitive effort; the peripheral route relies on heuristic cues or peripheral hints and imposes a relatively lower cognitive load. Li Zhong and Ma Jing [39] pointed out that users do not adopt a uniform information-processing mode; user involvement differences lead to different information processing routes.

In online health communities, patients face a large amount of heterogeneous information about physicians, and their processing methods vary with individual differences and contextual features. The Elaboration Likelihood Model has been widely applied to studies of online health behaviour to explain how users make decisions when confronted with complex health information [40–43]. Compared with the technology acceptance model, which focuses only on perceived usefulness and ease of use, the Elaboration Likelihood Model explains the mechanisms for processing complex health information. Compared with the unified theory of acceptance and use of technology, which considers multiple factors but lacks a process perspective, the Elaboration Likelihood Model provides a theoretical framework for understanding information-processing procedures. Jin Xiaoling et al. [41] showed that, for users of health-related public accounts, information quality on the central route and source credibility on the peripheral route exert more potent effects on liking intention than on commenting intention. Wei Wu and Xie Xingzheng [44] demonstrated that product content quality on the central route positively influences the need satisfaction of users who pay for online knowledge, thereby promoting their intention to continue paying. These studies confirm the model’s effectiveness in explaining online behaviour. However, an information-processing framework alone remains insufficient in the highly uncertain and risk-sensitive context of patients’ medical choices. Patients must process a large volume of information and, at the same time, cope with uncertainty about medical outcomes. In this context, whether patients trust physicians becomes the key link determining whether information processing truly translates into medical choice.

### Trust theory

Trust theory originates from organizational behaviour and social psychology. The integrated model proposed by Mayer et al. (1995) [12] defines trust as the willingness to be vulnerable based on positive expectations of the other party’s behaviour under risk conditions, and identifies ability, benevolence, and integrity as three core dimensions. Trust is a crucial mechanism for reducing risk and uncertainty in online medical settings characterized by high information asymmetry, intangible services, and uncertain outcomes. Prior studies have shown that transparency [45], physician reputation [46], and related value judgments [47] influence consumer trust, and that trust positively affects purchase intention [48].

In online medical environments, trust exhibits multiple dimensions. Beyond rational judgments based on ability and integrity, trust also includes affect-based trust grounded in emotional ties and institution-based trust relying on platform safeguards [49]. Research has found that trust often acts as a mediator in physician–patient relationships, linking physician reputation with patients’ consultation intentions [47]; at the same time, patients’ gift-giving or positive feedback strengthens other patients’ trust perceptions and generates a “trust transfer effect” [50].

In sum, existing research has confirmed the central role of trust in online health communities. However, most studies test its positive effect on intention overall and lack a systematic explanation of how different types of information further influence medical choice through the trust mechanism. Therefore, from the perspective of dual-route information processing, this study introduces the mechanism of trust to build a theoretical model of patients’ medical choice that better fits online health community scenarios.

## Research hypotheses and model

### Central route

According to the Elaboration Likelihood Model, the central route involves in-depth processing and critical thinking about information [51]. In online medical settings, information that requires patients to invest cognitive effort mainly includes the diagnostic and treatment content provided by physicians and the process of physician–patient interaction. Processing such information calls for specific medical knowledge and analytical ability, which aligns with the characteristics of the central route. Wang Yaqian and Cao Gaohui [52] found that article-quality features significantly influence the number of views through the central route. Therefore, content and interaction quality are appropriate central-route factors influencing patients’ online medical choices.

Content quality reflects the professionalism, completeness, and specificity of diagnostic and treatment advice provided by physicians. High-quality content contains substantive information relevant to diagnostic and treatment tasks and represents a core element of the central route. Wang Jianya et al. [53] and Feng Ying et al. [54] respectively confirmed the effects of paid knowledge content quality and information content quality on online trust and perceived utility. Li Qi et al. [55] reported that information quality on the central route shapes users’ cognitive attitudes in live-streaming commerce. In online medical environments, when patients carefully read physicians’ replies and assess their professionalism and specificity, high-quality diagnostic and treatment content strengthens their recognition of their professional competence and builds cognitive trust.

Interaction quality reflects the timeliness, continuity, and depth of physician–patient communication. Liao Chuhui and Chen Juan [56] found that interaction quality is key to user satisfaction and adoption intention. In online health communities, interaction characteristics such as response speed, reply frequency, and communication depth influence patient experience [57, 58]. When physicians respond promptly and in detail and maintain continuous communication, patients more readily build trust in physicians [59]. Therefore, interaction quality is a key factor for improving patients’ online trust in the context of online medical choice.

Hypotheses:

- H1:Content quality positively influences patients’ online trust in the context of online medical choice.
- H2: Interaction quality positively influences patients’ online trust in the context of online medical choice.

### Peripheral route

Compared with the complex and abstract information on the central route, information on the peripheral route is more straightforward. Studies have shown that when users lack advanced cognition and analytical ability, they actively choose the peripheral route, which requires less time and effort [60]. In this process, the credibility and professionalism of the target object serve as the primary influencing factors [61, 62]. Therefore, reputation credibility, which reflects patients’ evaluations of physicians’ service quality, and professional competence credibility, which represents physicians’ medical knowledge and skills, are reasonable peripheral-route factors that influence patients’ online trust in the context of online medical choice.

First, online word of mouth serves as an effective signal of reputation credibility. Wang Y. et al. [63] found that physicians’ word of mouth is a key factor influencing patients’ decision to seek consultation. Research has shown that users often consult online word of mouth before making purchase decisions [64], and electronic word of mouth significantly shapes decision-making behaviour [65]. In medical services, evaluations and recommendations from other patients represent collective experience and quickly establish initial trust. Electronic word of mouth as a proxy for reputation credibility is often measured by the number of online comments [66] and the popularity of recommendations [67].

Second, physicians’ certifications reflect their professional competence and credibility [13]. When information is insufficient, patients prefer to trust more reliable signals, such as job titles that meet national standards [68]. Although physicians’ credentials, including job titles, degrees, and years of practice, cannot directly reflect their clinical competence, these institutionalised certification signals quickly establish preliminary trust. However, existing studies have suggested that in digital health and online health information contexts, users with higher medical scepticism perceive a weaker influence of formal credentials on trust [69]. A mixed-method study in Saudi Arabia also reported that most public members rely more on the website or publication reputation and content consistency with existing knowledge than on authors’ titles or degrees when evaluating the credibility of health information [70]. In addition, some reports have indicated that younger people express doubts about the actual reliability of official or institutional credentials; even when physicians hold titles or certifications, concerns about transparency or trustworthiness may prevent these credentials from being fully trusted [71]. Therefore, while researchers theoretically regard professional competence credibility as an important peripheral-route factor, its practical influence remains uncertain.

Finally, service capability credibility reflects physicians’ activity level and service commitment on the platform. The frequency service providers update service content influences users’ purchase intentions [72]. Physicians’ indicators on the platform, such as knowledge contributions, number of popular science articles, and timeliness of replies, demonstrate continuity and reliability of services [73–75]. These peripheral cues strengthen patients’ trust in physicians’ service capability. However, similar to professional credentials, their effect may diminish as patients increasingly emphasise dynamic signals such as actual interaction and feedback from other users.

Hypotheses:

- H3: Reputation credibility positively influences patients’ online trust.
- H4: Professional competence credibility positively influences patients’ online trust.
- H5: Service capability credibility positively influences patients’ online trust.

### Mediating variable

Online trust refers to patients’ psychological state in uncertain online medical environments, where they hold positive expectations about physicians’ ability, benevolence, and integrity, are willing to take risks, and rely on physicians to provide medical services [47]. The particularity of medical services makes trust the key bridge that connects information perception with behavioural decision-making. Based on the model of online trust formation proposed by McKnight et al. [49], trust forms through an integrated process involving multiple mechanisms. Knowledge-based trust forms when patients thoroughly understand medical content and corresponds to the central route; calculus-based trust forms when patients quickly judge external signals and corresponds to the peripheral route. Patients analyse professional information physicians provide through the central route and form cognitive trust; through the peripheral route, patients rely on others’ evaluations, physicians’ credentials, and similar peripheral cues to quickly build initial trust.

Trust-transfer theory states that trust transfer occurs when a trustor’s perceived trust in a trustee derives from the trust perceptions of a third party [76]. In online medical contexts, patients rely on social signals such as other patients’ evaluations and gift-giving to make trust judgments. This study uses the number of gifts to measure online trust. This culturally specific indicator is standard on Chinese online medical platforms, where patients present virtual gifts to physicians to express gratitude and trust. It directly reflects patients’ trust and satisfaction with physicians [77].

Existing research has demonstrated the mediating role of trust in online medical decision-making. Huang Sihao et al. [78] found that perceived trust positively influences consumers’ continuous purchase intentions. Mou and Cohen showed that patients’ trust in online health services significantly shapes their usage intentions in the healthcare context. Therefore, stimulation by dual-route information in online health communities builds patients’ online trust, promoting online medical choice behaviour. Some studies also define online trust as users’ overall evaluation of the utility of a product or service after weighing perceived benefits against the costs of obtaining it [79, 80]. Dongwei Yan et al. [81] found that customers’ green online trust enhances sustainable consumption intentions. Hence, dual-route information in online health communities stimulates patients’ online trust in medical choice, and online trust further promotes online medical choice behaviour.

Hypothesis:

- H6: Patients’ online trust positively influences online medical choice behaviour.

### Moderating variables

Motivation and ability are the main moderators when choosing between the central and peripheral routes. Motivation consists of the need for cognition and involvement; ability refers to users’ cognitive level. User involvement in information processing reflects both motivation and ability [82]. The Elaboration Likelihood Model suggests that highly involved users process information through the central route and carefully evaluate information quality. In contrast, low-involvement users rely more on peripheral cues to make judgments. Chen Yitao [83] found that involvement moderates the effect of high-quality content on transactional psychological contracts and the effect of anchor trust on relational psychological contracts. Ke Qing et al. [84] showed that user involvement moderates the effects of information quality and source credibility on persuasive outcomes. Disease severity and urgency influence patients’ involvement in medical decision-making, shaping their information-processing strategies and trust-formation routes.

Hypotheses:

- H7a: User involvement positively moderates the effects of central-route factors on online trust.
- H7b: User involvement negatively moderates the effects of peripheral-route factors on online trust.

Online medical choice behaviour refers to the process in which users evaluate service attributes and make rational decisions, namely, purchasing services that meet specific needs at the lowest possible cost [85]. For users in online health communities, the decision process requires selecting suitable physicians and consulting according to their payment capacity [86]. However, the direct effect of price on medical choice is not always significant [46] and often operates by moderating the relationship between trust and behaviour.

Hypothesis:

- H8: Consultation price negatively moderates the effect of online trust on online medical choice behaviour.

**Fig. 1:**
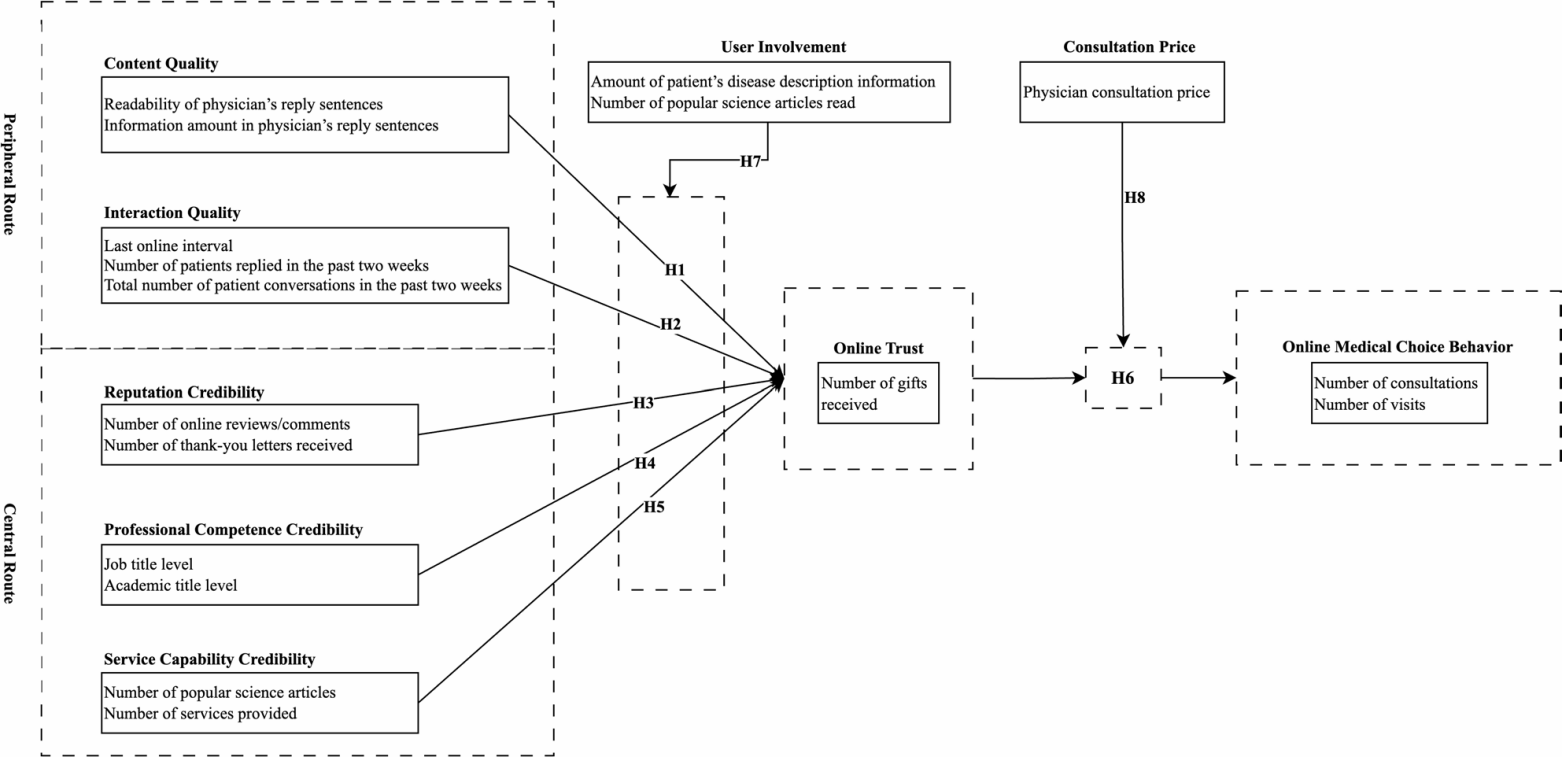
Research model

In summary, drawing on the Elaboration Likelihood Model and trust theory, we consider patients’ medical choice as a process in which external information stimuli influence final decisions through the mechanism of trust. External stimuli consist of two aspects: the central route, represented by the information quality of consultation content, and the peripheral route, represented by physicians’ credibility cues. Online trust serves as a mediator linking information perception with medical choice behaviour. We treat user involvement as a moderator between the information-processing route and online trust, and consultation price as a moderator between online trust and patients’ online medical choice behaviour. Fig. 1 presents the research model.

## Research design

### Data collection

The data used in this study came mainly from the psychiatry and psychology department of the online health community “Haodf.com.” This department primarily addresses depression, anxiety, and bipolar disorder, where treatment relies mainly on psychological counselling. These conditions exhibit chronic courses, long cycles, and frequent recurrence, which increase patients’ dependence on physicians’ consultations. This department features relatively similar symptom spectra with some overlaps compared with other departments. The department includes over 2,000 recommended physicians, of whom over 1,000 provide online consultation services. The personal webpages of these physicians receive about 500 visits per day. Under the premise of relatively comparable patient motivations, this department provides broad sample coverage and satisfies the “heterogeneity based on homogeneity” requirement in statistical analysis. Therefore, we selected patients from this department as our study subjects.

**Fig. 2:**
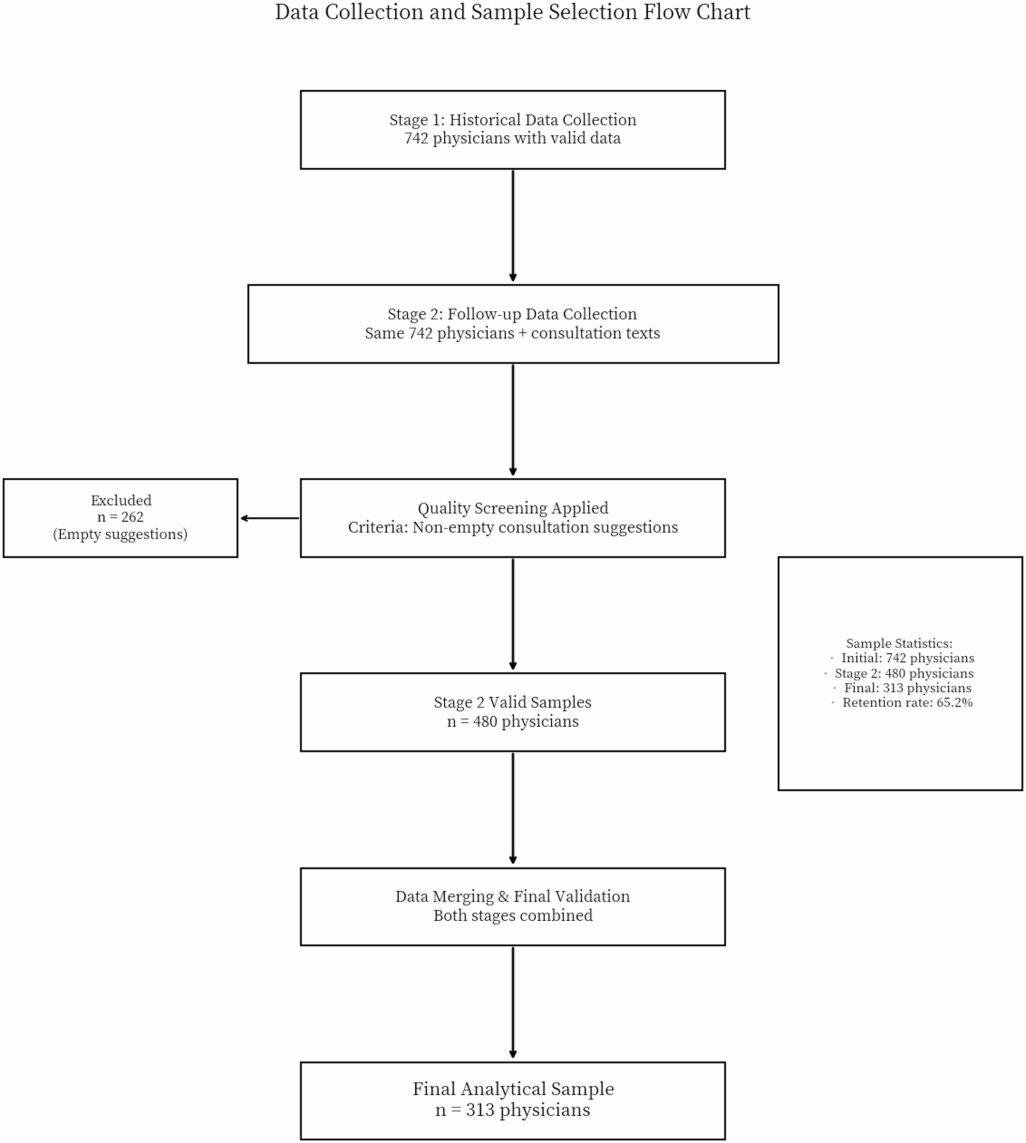
Data collection and processing

We collected the data in two stages. In the first stage, we retrieved structured information for all physicians in the psychiatry and psychology department for whom historical data were available. Because of platform anonymisation restrictions, we merged patient data at the physician level and analysed users’ behaviours corresponding to each physician. After preprocessing, we obtained 742 valid physician samples. In the second stage, we followed the same 742 physicians, collected the same structured indicators, and supplemented the data with consultation text information from physician–patient interactions (such as patients’ disease descriptions, physicians’ diagnostic and treatment suggestions, and the number of dialogues between physicians and patients). Using “non-empty physician consultation suggestions” as the screening criterion, we obtained 480 valid samples. By merging the samples from both stages, we arrived at 313 valid samples. Fig. 2 illustrates the original data collection process.

### Variable design

This study investigates how dual-route factors influence patients’ online medical choice decisions. Table 1 presents the descriptions of all variables. The dependent variable is patients’ online medical choice behaviour, which we measured using the incremental number of consultations and the incremental number of visits within a fixed time window. These measures reflect physicians’ attractiveness and patients’ choice behaviour on the platform. We applied the same time window to all sample collection stages to ensure comparability across different stages. The independent variables include physicians’ interaction quality, content quality, reputation credibility, professional competence credibility, and service capability credibility. Online trust is the mediating variable, and user involvement and consultation price are the moderating variables. In the following section, we describe the indirectly measured variables: content quality, interaction quality, and user involvement.

**Table 1:** Research variables

Category	Latent Variable	Observation variable	Express
Central Route (CR)	Content Quality (CQ)	Readability of physician’s reply sentences	Text quantization
		Information amount in physician’s reply sentences	Text quantization
	Interaction Quality (IQ)	R - Last online interval	Indirect statistics
		F - Number of patients replied in the past two weeks	Indirect statistics
		M - Total number of patient conversations in the past two weeks	Indirect statistics
Peripheral Route (PR)	Reputation Credibility (RC)	Number of online comments	Direct representation
		Number of thank-you letters received	Direct representation
	Professional Competence Credibility (PCC)	Job title level	Assignment (1–5)
		Academic title level	Assignment (1–5)
	Service Capability Credibility (SCC)	Number of popular science articles	Direct representation
		Number of services provided	Direct representation
Moderator	User Involvement (UI)	Amount of patient’s disease description information	Text quantization
		Number of popular science articles read	Direct representation
	Consultation Price (CP)	Physician consultation price	Direct representation
Mediator	Online Trust (OT)	Number of gifts received	Direct representation
Dependent Variable	Online Medical Choice Behaviour (OMCB)	Number of consultations	Direct representation
		Number of visits	Direct representation

1. Content quality
  Following the approach of Cao et al. [87] on quantifying consultation interactions, we measured content quality using readability and information amount. Readability refers to how easily patients can read and understand the physician’s reply, and researchers measure it using the average word frequency or the number of characters per sentence [88]. Information amount refers to the quantity of relevant information in the physician’s reply, typically measured by the number of non-repetitive keywords [87]. In this study, we defined content quality as the extent to which physicians’ online replies enhanced patients’ understanding of their conditions, operationalised as readability (defined as text length) and information amount (defined as the number of keywords). We quantified every reply text for each physician, then calculated the average across all replies as the content quality score. The quantification process involved four steps: ① segmenting each reply text into words; ② calculating word frequency, where the total count of words served as the readability score; ③ removing stop words; and ④ calculating the number of unique words representing the information amount.
2. Interaction quality
  The RFM theory evaluates user engagement by measuring recency (R), frequency (F), and monetary value (M), which capture the positivity and contribution of user behaviour. This framework is widely applied to describe interaction quality [89]. In online physician–patient interactions, the recency of physicians’ services represents R, F by the frequency of physicians’ services, and M by the depth of physician–patient interactions.
3. User involvement
  The moderating variable of user involvement refers to patients’ level of participation and cognitive engagement in online consultations. Based on cognitive involvement theory and information processing theory, highly involved patients are likelier to engage in deeper information processing and show stronger initiative and cognitive investment in health-related behaviours [87, 90]. We measured user involvement from two dimensions. We assessed information acquisition involvement using the number of popular science articles patients read, which reflects their proactive acquisition of health-related information and cognitive participation. We evaluated expression involvement by the length of patients’ textual descriptions of their diseases during consultations, calculated as the total number of punctuation marks. According to cognitive involvement theory, text length is a reliable indicator of cognitive involvement [90]; longer descriptions indicate higher cognitive investment, reflecting patients’ deeper thinking and more precise articulation of their health conditions.

### Model construction

We chose regression analysis to test our hypotheses because our study focuses on estimating direct effects and moderating effects among observed variables, and this approach has been widely adopted in research on online health communities [91]. Based on the theoretical analysis and research hypotheses, we constructed the following regression models for empirical testing:

1. Model a: Effect of dual-route factors on online trust
  Online trust_i_ = β₀ + β₁ content quality_i_ + β₂ interaction quality_i_ + β₃ reputation credibility_i_ + β₄ professional competence credibility_i_ + β₅ service capability credibility_i_ + ε_i_
2. Model b: Effect of online trust on online medical choice behaviour
  Online medical choice behaviour_i_ = α₀ + α₁ online trust_i_ + ε_i_
3. Model c: Moderating effect of user involvement
  Online trust_i_ = γ₀ + γ₁ central route_i_ + γ₂ peripheral route_i_ + γ₃ user involvement_i_ + γ₄ (central route_i_ × user involvement_i_) + γ₅ (peripheral route_i_ × user involvement_i_) + ε_i_
4. Model d: Moderating effect of consultation price
  Online medical choice behaviour_i_ = δ₀ + δ₁ online trust_i_ + δ₂ consultation price_i_ + δ₃ (online trust_i_ × consultation price_i_) + ε_i_

We log-transformed all continuous variables as Y = ln(X + 1) to reduce heteroscedasticity and improve normality. Before testing moderating effects, we mean-centred the relevant variables to reduce multicollinearity.

## Empirical analysis and results

### Descriptive statistics and correlation analysis

Before conducting the empirical analysis, we performed descriptive statistical analysis on the primary research variables (see Table 2). The results show that all latent variables and their observation indicators exhibit reasonable variability and distribution across the sample. It is important to note that we log-transformed all variables to reduce the influence of extreme values and improve normality. In addition, we aggregated the observation indicators into their corresponding latent variables according to theoretical constructs, including content quality, interaction quality, reputation credibility, professional competence credibility, service capability credibility, user involvement, online trust, and online medical choice behaviour. Overall, the variables displayed moderate variation, providing a solid data foundation for the subsequent regression analyses and tests of moderating effects.

**Table 2:** Descriptive statistics of the variable

Variable	Obs	Mean	Std. dev.	Min	Max
CQ	313	5.972	1.054	4.342	7.316
IQ	313	8.637	3.525	0.693	14.583
RC	313	4.027	3.278	0.000	10.487
PCC	313	2.315	0.662	0.693	3.219
SCC	313	4.213	2.123	1.386	8.925
UI	313	14.930	2.913	1.582	18.105
CP	313	4.974	0.293	4.007	5.680
OT	313	2.452	1.274	0.693	5.118
OMCB	313	16.955	2.624	10.815	21.825

(1) Variable abbreviations are as follows: *CQ* Content quality, *IQ* Interaction quality, *RC* Reputation credibility, *PCC* Professional competence credibility, *SCC* Service capability credibility, *UI* User involvement, *CP* Consultation price, *OT* Online trust, *OMCB* Online medical choice behaviour. The same abbreviations apply hereafter

(2) All variables were log-transformed using the Y = ln(X) formula

The results of the correlation analysis in Table 3 indicate that the correlation coefficients among the core explanatory variables fall within a reasonable range, with significance levels consistent with expectations, and no evidence of high correlations. Further collinearity diagnostics show that all variance inflation factor (VIF) values are far below the commonly used threshold of 10, with an average VIF of only 1.06. These results confirm that the model does not suffer from significant multicollinearity. Therefore, the data structure of this study is robust and suitable for supporting the subsequent regression and moderation analyses.

**Table 3:** Correlation analysis and collinearity diagnosis

No.	Variable	1	2	3	4	5	6	7	8	VIF
1	CQ	1.000								1.03
2	IQ	−0.068	1.000							1.09
3	RC	0.021	−0.055	1.000						1.09
4	PCC	0.062	0.036	0.041	1.000					1.04
5	SCC	−0.009	0.073	0.005	−0.107*	1.000				1.03
6	UI	0.002	0.157***	0.126**	0.107*	0.016	1.000			1.06
7	CP	0.038	−0.069	−0.018	0.025	0.026	−0.015	1.000		1.01
8	OT	0.133**	0.189***	0.232***	−0.042	0.105*	0.138**	0.038	1.000	1.16
9	OMCB	0.082	0.143**	0.186***	−0.023	0.126**	0.048	−0.043	0.345***	-

Mean VIF = 1.06

***p < 0.01, **p < 0.05, *p < 0.1; values without marks are insignificant

### Regression results

Table 4 presents the regression results. In Model a, the dependent variable is online trust. First, regarding the central route, content quality (β = 0.177, *p* < 0.01) and interaction quality (β = 0.075, *p* < 0.001) both exert significant positive effects on patients’ online trust, thus supporting Hypotheses H1 and H2. The coefficient for content quality is higher, suggesting that patients rely more on the completeness and readability of information when forming trust. Although interaction quality also has a significant impact, its effect is weaker, possibly because interaction information requires patients’ experience to become more effective.

**Table 4:** Regression results

Variable	Model a Coefficient (SE)	95% CI	Model b Coefficient (SE)	95% CI	Hypothesis Test Results
CQ	0.177*** (0.065)	[0.049, 0.304]			H1 Supported
IQ	0.075*** (0.019)	[0.037, 0.113]			H2 Supported
RC	0.094*** (0.021)	[0.053, 0.134]			H3 Supported
PCC	−0.114 (0.103)	[−0.319, 0.090]			H4 Not Supported
SCC	0.050 (0.032)	[−0.013, 0.114]			H5 Not Supported
OT			0.710*** (0.110)	[0.494, 0.925]	H6 Supported
Constant	0.421 (0.511)	[−0.585, 1.425]	15.215*** (0.303)	[14.619, 15.811]	-
R²	0.126		0.119		-
F	8.85***		41.90***		-

Second, regarding the peripheral route, reputation credibility (β = 0.094, *p* < 0.001) shows a significant positive effect on online trust, supporting Hypothesis H3. In contrast, professional competence credibility (β = − 0.114, *p* >0.1) and service capability credibility (β = 0.050, *p* >0.1) are not significant, and therefore Hypotheses H4 and H5 are not supported. Several factors may explain professional competence, credibility, and service capability insignificance. First, patients’ reliance on professional title levels has decreased in online channels where information is rich and diverse, reducing the influence of competence-related credibility on online trust [36]. Second, the virtual nature of online medical choice increases patients’ perceived risk, and online word of mouth—widely recognised by the public—effectively mitigates this risk. At the same time, competence-related credibility plays a minor role. Third, since patients’ usage of online health communities is still far below that of e-commerce platforms, the text-based consultation model already satisfies most patients’ needs. In contrast, telephone or video consultations are relatively more expensive and less widely adopted, leaving room for growth. Therefore, the number of service types reflecting physicians’ breadth of effort currently does not significantly influence online trust [92].

Finally, in Model b, the dependent variable is online medical choice behaviour. The results indicate that online trust significantly positively affects online medical choice behaviour (β = 0.541, *p* < 0.001), thus supporting Hypothesis H6. This finding demonstrates that online trust is crucial in patients’ final medical choice decisions. When patients establish a higher level of trust, their willingness to select physicians for consultation significantly increases.

### Moderating effect tests

We first mean-centred the relevant variables to examine the moderating effects of user involvement and consultation price in patients’ medical choice processes. Then we constructed regression models by introducing user involvement (Model c) and consultation price (Model d) as moderators.

As shown in Table 5, Model c indicates that the interaction between user involvement and the central route significantly positively affects online trust (β = 0.014, *p* < 0.1), supporting Hypothesis H7a. However, the interaction term between user involvement and the peripheral route is insignificant (β = −0.003, *p* > 0.1), and Hypothesis H7b is not supported. The characteristics of peripheral-route information may explain this result: it is typically highly structured, straightforward, and easy to understand, so patients can perceive its value without investing substantial cognitive resources. Therefore, differences in user involvement do not significantly alter its effect on online trust. In contrast, central-route information is often more complex and requires deeper cognitive processing, which explains why user involvement exerts a more substantial moderating effect in this path.

**Table 5:** Regression results of the moderating effect

Variable	Model c	95% CI	Model d	95% CI	Hypothesis test results
Constant	−0.233 (0.508)	[−1.233, 0.765]	17.116*** (2.400)	[12.393, 21.838]	-
CR	0.073*** (0.019)	[0.036, 0.111]			-
PR	0.074*** (0.017)	[0.040, 0.108]			-
UI	0.055* (0.029)	[−0.002, 0.111]			-
CR × UI	0.014* (0.007)	[−0.001, 0.028]			H7a supported
PR × UI	−0.003 (0.006)	[−0.016, 0.009]			H7b not supported
OT			0.717*** (0.109)	[0.502, 0.932]	-
CP			−0.384 (0.481)	[−1.330, 0.562]	-
OT × CP			−0.620* (0.359)	[−1.326, 0.087]	H8 supported
R²	0.123		0.130		-
F	8.60***		15.42***		-

Variable abbreviations are *CR* Central route, *PR* Peripheral route

Model d tested the moderating role of consultation price in the relationship between online trust and online medical choice behaviour. The results show that consultation price has a significant adverse moderating effect (β = −0.620, *p* < 0.1), supporting Hypothesis H8. This finding indicates that higher consultation prices weaken the positive impact of online trust on online medical choice behaviour.

**Fig. 3:**
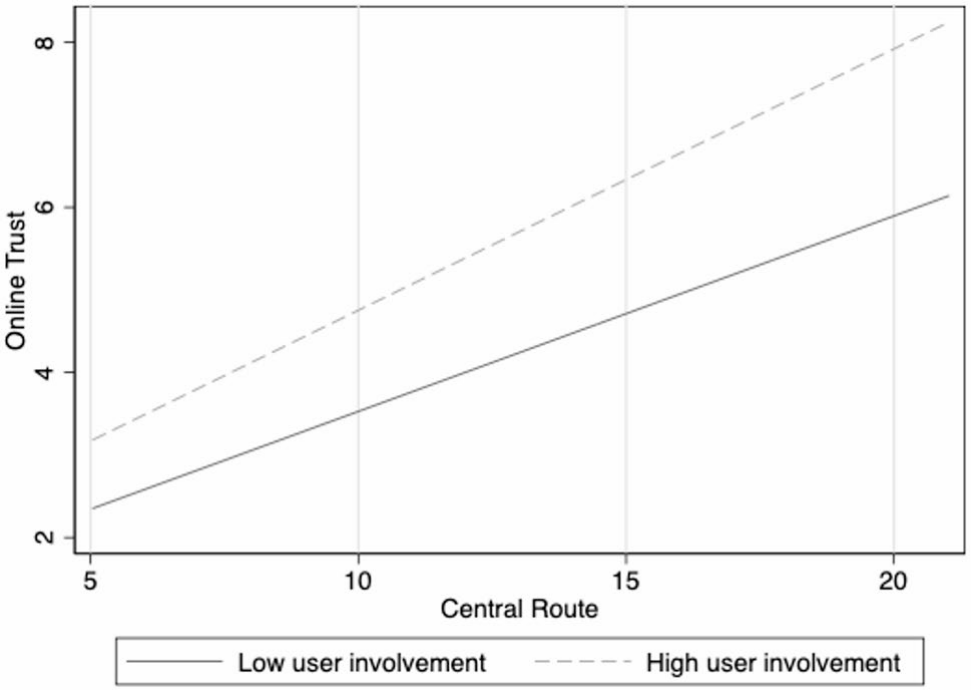
Moderating effect of user involvement in the central route

**Fig. 4:**
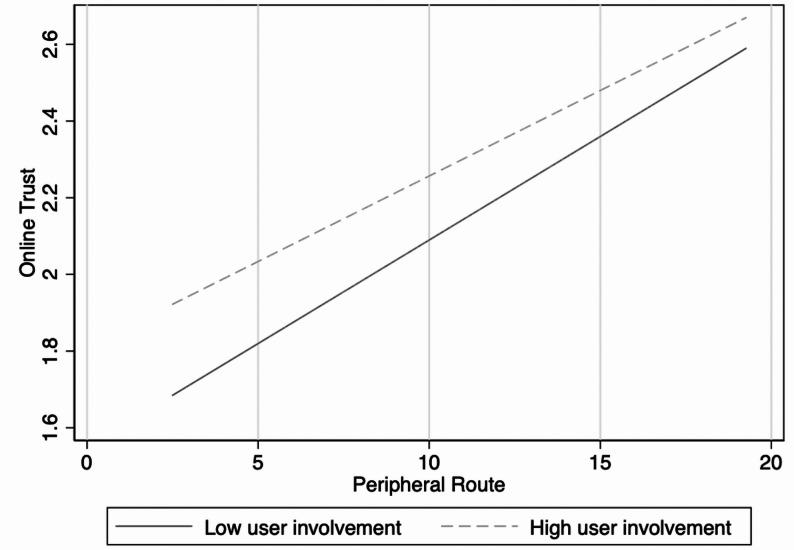
Moderating effect of user involvement in the peripheral route

**Fig. 5:**
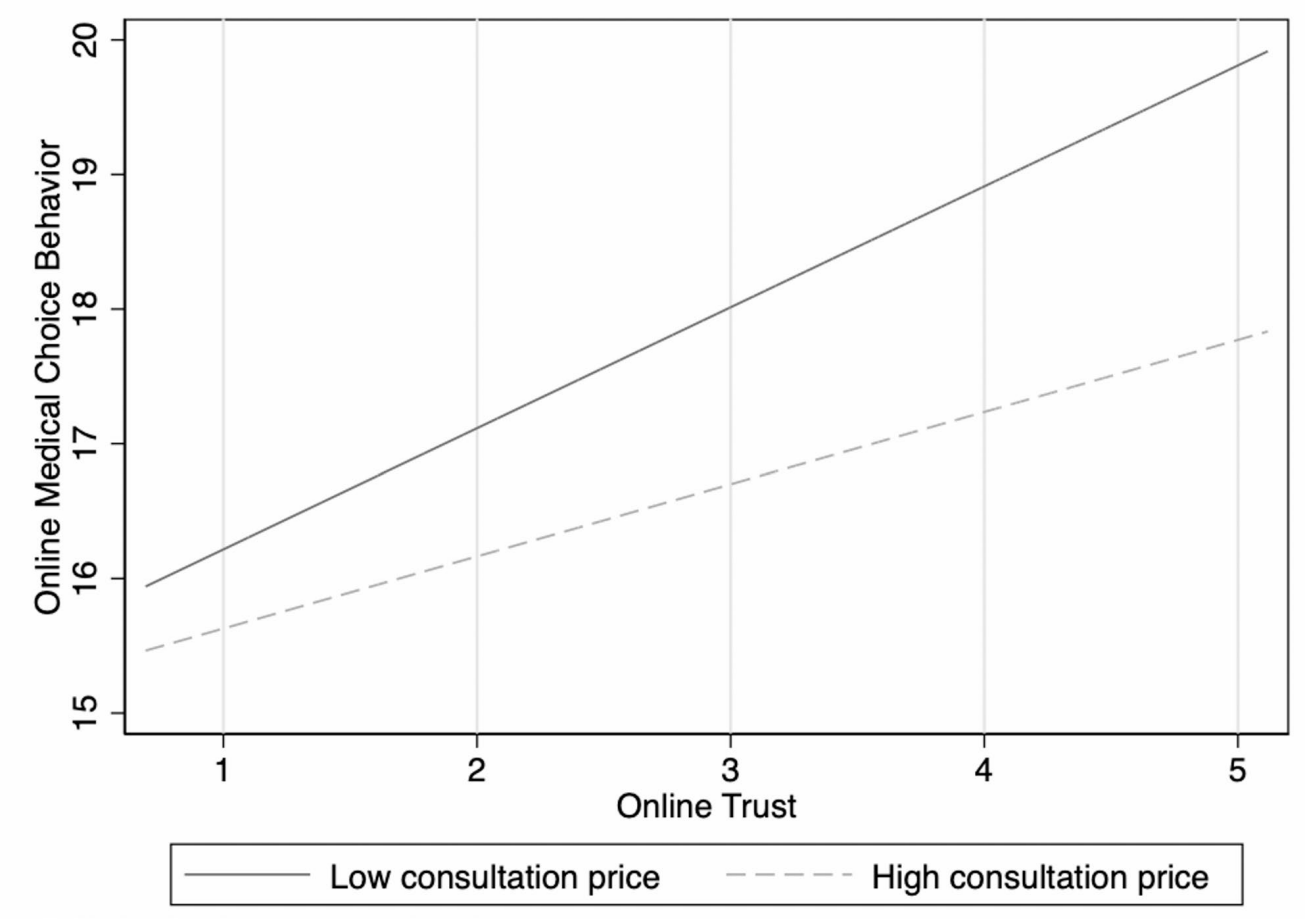
Moderating effect of consultation price

Figures 3, 4 and 5 present the results of the moderation effect tests. First, user involvement significantly moderates the relationship between the central route and online trust (Fig. 3). Specifically, under the condition of high user involvement, the positive impact of the central route on online trust was more substantial, with a steeper regression slope than under low user involvement. This indicates that when patients invest more cognitive resources during information processing, they are more likely to strengthen their trust in physicians through central-route information. At the same time, user involvement showed a significant positive moderating effect on the relationship between the peripheral route and online trust (Fig. 4). Compared with the low involvement condition, the peripheral route more strongly enhanced online trust under the high involvement condition. This suggests that even heuristic cues and peripheral information can be more effectively transformed into perceived trust when patients actively engage. Finally, consultation price exerted a significant adverse moderating effect on the relationship between online trust and online medical choice behaviour (Fig. 5). Under the low consultation price condition, online trust’s positive effect on online medical choice behaviour was more substantial. By contrast, this effect was significantly weakened under the high consultation price, suggesting that high prices reduce the extent to which patients convert trust into actual medical choice behaviour.

In summary, user involvement strengthened the formation of online trust under the influence of both central and peripheral-route information. In contrast, consultation price weakened the conversion of trust into medical choice behaviour.

### Robustness checks

To ensure the robustness of our findings, we applied several verification methods, including introducing control variables, using robust standard error estimation, and performing winsorization on the variables.

#### Regression results with controls

To examine the robustness of the research conclusions, we incorporated control variables into the original regression model, including the total number of dialogues, the total number of online days, and the overall popularity of recommendations, and then re-ran the regression analyses. As shown in Table 6, the results indicated that including control variables did not alter the core conclusions. Specifically, content quality (β = 0.160, *p* < 0.05), interaction quality (β = 0.073, *p* < 0.001), and reputation credibility (β = 0.097, *p* < 0.001) continued to exert significant positive effects on online trust. In contrast, professional competence, credibility, and service capability remained non-significant (*p* > 0.1). Thus, Hypotheses H1, H2, and H3 continued to receive support, while Hypotheses H4 and H5 were not supported. In addition, the interaction term between user involvement and the central route remained significantly positive (β = 0.013, *p* < 0.1), supporting Hypothesis H7a. In contrast, the interaction term with the peripheral route remained non-significant (*p* > 0.1), and Hypothesis H7b was not supported. Furthermore, online trust continued to significantly affect online medical choice behaviour (β = 0.709, *p* < 0.001), supporting Hypothesis H6. However, the moderating effect of consultation price on the relationship between online trust and online medical choice behaviour remained non-significant (*p* > 0.1), and Hypothesis H8 was not supported. Taken together, the results after adding control variables were consistent with those of the main model, further verifying the robustness of our conclusions.

**Table 6:** Regression results with controls

Variable	Model a	95%CI	Model b	95%CI	Model c	95%CI	Model d	95%CI
IQ	0.160** (0.066)	[0.031, 0.289]						
CQ	0.073*** (0.020)	[0.034, 0.111]						
RC	0.097*** (0.021)	[0.056, 0.138]						
PCC	−0.116 (0.104)	[−0.321, 0.090]						
SCC	0.050 (0.032)	[−0.014, 0.114]						
CR					0.056* (0.029)	[−0.001, 0.113]		
PR					0.070*** (0.019)	[0.032, 0.107]		
UI					0.076*** (0.017)	[0.041, 0.110]		
CR × UI					0.013* (0.007)	[−0.001, 0.028]		
PR × UI					−0.003 (0.006)	[−0.016, 0.009]		
OT			0.702*** (0.111)	[0.484, 0.920]			0.709*** (0.111)	[0.491, 0.926]
CP							−0.399 (0.490)	[−1.362, 0.565]
OT × CP							−0.620* (0.363)	[−1.335, 0.094]
TNC	−0.003 (0.028)	[−0.059, 0.052]	0.017 (0.057)	[−0.096, 0.129]	−0.004 (0.028)	[−0.059, 0.052]	0.017 (0.057)	[−0.096, 0.130]
TOD	−0.079 (0.171)	[−0.416, 0.259]	0.015 (0.351)	[−0.676, 0.707]	−0.079 (0.172)	[−0.417, 0.259]	−0.089 (0.355)	[−0.788, 0.609]
ORP	−1.047* (0.588)	[−2.203, 0.109]	−0.846 (1.207)	[−3.221, 1.530]	−1.103* (0.585)	[−2.254, 0.048]	−0.760 (1.205)	[−3.131, 1.612]
Constant	2.377** (1.167)	[0.080, 4.673]	16.414*** (2.073)	[12.335, 20.493]	1.720 (1.123)	[−0.490, 3.931]	18.497*** (3.155)	[12.290, 24.705]
R²	0.136		0.120		0.134		0.132	
F	5.98***		10.54***		5.88***		7.74***	

#### Regression results with robust standard errors

We further estimated the regression using robust standard errors to address potential heteroskedasticity issues. Table 7 shows the results: content quality, interaction quality, and reputation credibility all had significant positive effects on online trust (*p* < 0.001), while professional competence and service capability credibility remained non-significant, confirming the robustness of earlier conclusions. Meanwhile, online trust consistently and positively influenced online medical choice behaviour (*p* < 0.001). User involvement continued to significantly and positively moderate the relationship between the central route and online trust (*p* < 0.01), although its moderating effect on the peripheral route was not significant. The interaction between online trust and consultation price was insignificant, indicating that consultation price did not moderate the relationship between online trust and online medical choice behaviour in the robustness test. These findings show that the main hypothesis results remained robust after applying robust standard error estimation.

**Table 7:** Regression results with robust standard errors

Variable	Model a	95%CI	Model b	95%CI	Model c	95%CI	Model d	95%CI
CQ	0.177** (0.065)	[0.049, 0.305]						
IQ	0.075*** (0.020)	[0.036, 0.114]						
RC	0.094*** (0.021)	[0.052, 0.136]						
PCC	−0.114 (0.104)	[−0.320, 0.091]						
SCC	0.050 (0.033)	[−0.015, 0.115]						
UI					0.055** (0.018)	[0.019, 0.090]		
CP							−0.384 (0.485)	[−1.338, 0.570]
OT			0.710*** (0.097)	[0.519, 0.900]			0.717*** (0.096)	[0.529, 0.905]
CR × UI					0.014** (0.005)	[0.004, 0.024]		
PR × UI					−0.003 (0.005)	[−0.013, 0.006]		
OT × CP							−0.620* (0.315)	[−1.241, 0.000]
Constant	0.420 (0.498)	[−0.559, 1.400]	15.215*** (0.290)	[14.645, 15.785]	−0.234 (0.410)	[−1.042, 0.574]	17.116*** (2.479)	[12.237, 21.994]
R²	0.126		0.119		0.123		0.130	
F	9.05***		53.82***		12.49***		26.24***	

#### Robustness checks using winsorization

To minimise the influence of extreme values on regression results, we applied winsorization at the 1% and 99% levels for all variables. Table 8 reports that content quality, interaction quality, reputation credibility, and service capability credibility maintained significant positive effects on online trust, while professional competence credibility remained non-significant. Online trust consistently exerted a significant positive effect on online medical choice behaviour. Moreover, the moderating effect of user involvement on the central route remained significant, while its moderating effect on the peripheral route remained non-significant. In addition, consultation price demonstrated a significant adverse moderating effect on the relationship between online trust and online medical choice behaviour. Overall, the results remained consistent with the baseline regressions.

**Table 8:** Robustness checks with winsorized variables

Variable	Model1	95%CI	Model2	95%CI	Model3	95%CI	Model4	95%CI
CQ	0.176** (0.065)	[0.049, 0.303]						
IQ	0.076*** (0.019)	[0.038, 0.114]						
RC	0.094*** (0.021)	[0.054, 0.136]						
PCC	−0.116 (0.103)	[−0.319, 0.088]						
SCC	0.052 (0.033)	[−0.012, 0.116]						
UI					0.056* (0.029)	[−0.001, 0.112]		
CP							−0.399 (0.486)	[−1.356, 0.558]
OT			0.712*** (0.110)	[0.497, 0.928]			0.723*** (0.109)	[0.508, 0.938]
CR × UI					0.014* (0.007)	[−0.001, 0.028]		
PR × UI					−0.003 (0.006)	[−0.016, 0.009]		
OT × CP							−0.656* (0.372)	[−1.388, 0.076]
Constant	0.410 (0.510)	[−0.593, 1.412]	15.205*** (0.302)	[14.610, 15.800]	−0.270 (0.506)	[−1.267, 0.726]	17.177*** (2.426)	[12.403, 21.950]
R²	0.128		0.120		0.125		0.131	
F	9.01***		42.24***		8.79***		15.55***	

In summary, whether by introducing control variables, applying robust standard errors, or conducting winsorization, the core conclusions of this study did not undergo substantial changes, further confirming the reliability and robustness of the findings.

## Conclusions and implications

### Conclusions

Drawing on data from the psychiatry and psychology department of “Haodf.com” and combining the Elaboration Likelihood Model with signal theory, we systematically examined the effects of the central route and peripheral route on online trust through regression analyses, as well as the influence of online trust on patients’ online medical choice behaviour. We further investigated the moderating roles of user involvement and consultation price. The main conclusions are as follows:

First, a combination of multidimensional information from both the central and peripheral routes shapes patients’ online trust in medical decision-making, and this trust exerts a significant positive influence on their online medical choice behaviour. From the central route perspective, content quality and interaction quality significantly enhance online trust, reducing information asymmetry and strengthening patients’ perception of interaction. From the peripheral route perspective, reputation credibility is dominant, while service capability and professional competence credibility do not significantly affect perceived value. This finding indicates that in virtual medical environments, patients rely more on directly perceivable and verifiable information, such as interaction quality and online reputation, while being less sensitive to traditional credentials, such as professional titles. Possible reasons include: on the one hand, online medical choice emphasizes actual consultation experience and interactive feedback, so patients value information that directly reflects communication and service capabilities; on the other hand, the uncertainty and perceived risk in virtual consultation contexts drive patients to rely more heavily on historical feedback and word of mouth from other patients, thereby enhancing their trust in physicians.

Second, user involvement positively moderates the effect of central-route information on online trust, whereas consultation price negatively moderates the relationship between online trust and online medical choice behaviour. However, user involvement does not significantly moderate the relationship between peripheral-route information and online trust. The underlying reason is that central-route information is more complex and contains higher information content, requiring patients to engage in analytical thinking to assess the logic and relevance of such factual information, thereby identifying reliable and valid evidence. In contrast, peripheral-route information is more straightforward and summarised by the broader public, enabling patients to judge its reliability quickly. Thus, processing central-route information requires higher user involvement to transform cognition into online trust, while peripheral-route information depends less on patients’ involvement and cognitive effort.

### Theoretical contributions

(1) We constructed a dual-pathway mechanism model of patients’ online medical choice from the information processing perspective. Previous studies often relied on single dimensions, such as the role of trust [36] or physician reputation [32]. Although these studies revealed some key factors, they generally exhibited fragmented and one-dimensional perspectives. Based on the central route and peripheral route of ELM, this study systematically integrated content quality and interaction quality (central route) with reputation credibility, professional competence credibility, and service capability credibility (peripheral route), while introducing online trust as a mediator, to build a complete dual-pathway mechanism. This design addressed the limitations of previous fragmented approaches and explained how different types of information influence trust through distinct processing routes, thereby affecting online medical choice behaviour. This perspective also echoes the dual-route information processing framework of heuristic–systematic processing proposed by Gao et al. [93].

(2) We deepened the understanding of the mediating role of online trust in medical choice. Although existing studies widely recognise the importance of trust in physician–patient relationships, most only examine its direct effect on consultation intention or payment intention [47], without analysing how different types of information operate through trust mechanisms. For example, some research has shown that physician reputation significantly influences patients’ medical choice intention [36, 38]. However, researchers often simplify trust into a single latent variable without distinguishing its role across different information pathways. The contribution of this study lies in revealing that central-route information (such as content quality and interaction quality) relies more on rational processing and affects patients’ cognitive-based trust. In contrast, peripheral-route cues (such as reputation credibility) are more associated with heuristic judgment and calculative trust. This finding extends McKnight et al.’s [49] model of online trust formation into the medical context. It fills the gap in prior literature that lacked systematic explanations of the “information–trust–behaviour” transmission mechanism. It thus provides new evidence for understanding how different types of information transform into medical choice behaviour.

(3) We revealed user involvement and consultation price boundary conditions in medical decision-making. Prior studies on medical choice mainly focused on physicians’ characteristics, reputation, or service indicators [7, 34], while rarely incorporating individual motivation and contextual constraints into the same framework. Our study found that user involvement significantly strengthened the effect of central-route information on trust but did not significantly influence peripheral-route information. At the same time, the consultation price weakened the relationship between trust and medical choice. Unlike the macro-level conclusion in previous research that “patient heterogeneity leads to different medical choice preferences” [33, 37], our findings more specifically demonstrate how individual motivation and economic cost alter information processing pathways and the trust transmission chain. This contribution extends discussions within ELM theory about individual differences and situational variables [39, 44]. It provides a more fine-grained explanation for medical choice research, highlighting the importance of considering moderating variables in high-risk medical decision-making.

### Managerial implications

Based on the findings of this study on dual-path medical choice behaviour, we propose several managerial implications to optimise patients’ online consultation experience and promote the sustainable development of online medical communities:

(1) Strengthen the centrality of reputation credibility in platform interface design to enhance patient trust and conversion. The results indicate that reputation credibility (number of online reviews and thank-you letters) under the peripheral route significantly affects online trust. In contrast, professional competence credibility (job title, academic title) and service capability credibility (number of services and popular science articles) do not show significant effects. This suggests that patients rely more on directly perceivable feedback from other patients rather than professional qualifications or service counts. Therefore, platforms should prioritise the visualisation of reputation information on physicians’ homepages, highlighting the incremental growth of thank-you letters, the trend of patient reviews, and the distribution of word-of-mouth evaluations through clear numerical indicators and graphical designs. In contrast, traditional indicators such as titles, degrees, and service quantities can be placed in secondary display areas, thereby reducing information overload and improving the efficiency of trust transmission.

(2) Improve service quality mechanisms toward response timeliness to facilitate trust building. The findings reveal that interaction quality significantly promotes online trust, with physicians’ response speed playing a particularly critical role. Platforms should therefore develop a multi-level response monitoring and incentive system. For example, a real-time response alert can remind physicians when they remain offline or fail to reply for extended periods; a “response timeliness index” can be established to classify and publicly display average response times; doctors with faster responses may receive priority in search results and traffic allocation; and the platform can introduce a “quick response” badge, enabling patients to easily identify physicians who engage in timely interactions, thereby shortening the trust formation process.

(3) Promote differentiated recommendations and dynamic pricing strategies based on user involvement and price sensitivity. The results demonstrate that user involvement positively moderates the effect of central route information on online trust. In contrast, consultation price negatively moderates the conversion of trust into online medical choice behaviour. Platforms can apply natural language processing to assess patients’ level of involvement from the length and information density of disease descriptions, supplemented by reading behaviours of popular science articles, and generate real-time involvement scores. Based on this assessment, highly involved patients should be recommended to physicians with strong interaction quality and professional content, even if the consultation price is higher; in contrast, patients with lower involvement are better matched with physicians with good reputation credibility and moderate pricing, thus lowering decision barriers. In addition, platforms may explore dynamic pricing mechanisms that integrate service ratings, response indices, and patient involvement levels to optimise the transformation of trust into online medical choice behaviour.

## Limitations and future research

This study has several limitations. First, the sample is limited to psychiatrists on the “Haodf.com” platform, leading to a restricted sample size and a lack of diversity, limiting the findings’ external validity and cross-cultural generalizability. Second, regarding measurement tools, we used disease description length as a proxy for user involvement without direct validation against standardised scales. This indicator primarily captures cognitive engagement but may fail to reflect emotional and behavioural involvement, and individual expression habits and cultural background may fully influence text length. In addition, using the number of gifts as a proxy for online trust and the number of thank-you letters for reputation credibility may introduce politeness bias, thus insufficiently capturing both cognitive and affective dimensions of trust. Similarly, measuring content quality only through text length and keyword density does not fully represent important aspects of medical communication, such as empathy and clarity. Third, we did not incorporate patient-specific characteristics (e.g., gender, personality) into the model, which may influence medical choice behaviour. Moreover, the non-significant moderation effect of user involvement on peripheral-route information could be due to limited statistical power, as the current sample size may not be sufficient to detect minor effects.

Future research should address these issues in several ways. First, the study will be extended to multiple online health platforms (e.g., Zocdoc, Doctolib) and different medical specialties to conduct cross-cultural validation and improve generalizability. Second, mixed-method designs should be adopted that integrate survey data with behavioural indicators such as time spent on physician pages and consultation frequency to capture both cognitive and emotional dimensions of online trust and multiple dimensions of user involvement. Third, linguistic measures such as empathy expressions and clarity (e.g., using natural language processing tools like LIWC) should be incorporated to assess content quality better and align with the medical communication literature. Fourth, the model should include the patient and physician’s individual characteristics to explore their direct effects and moderating roles in medical choice behaviour. Finally, we will conduct power analyses to determine appropriate sample sizes and ensure the ability to detect theoretically meaningful effects.

## Data Availability

The datasets generated and analysed during the current study are available in the Baidu Pan repository, https://pan.baidu.com/s/1QWL3EYkLLrSMUL5LIArEFQ?pwd=q8gg.

## Abbreviations

CNNIC: China Internet Network Information Centre
CP: Consultation price
CQ: Content quality
CR: Central route
ELM: Elaboration Likelihood Model
IQ: Interaction quality
OMCB: Online medical choice behaviour
OT: Online trust
PCC: Professional competence credibility
PR: Peripheral route
RC: Reputation credibility
RFM: Recency–Frequency–Monetary (framework)
RQ: Research question(s)
SCC: Service capability credibility
UI: User involvement
VIF: Variance inflation factor
